# Single-center prospective study on the efficacy of nivolumab against platinum-sensitive recurrent or metastatic head and neck squamous cell carcinoma

**DOI:** 10.1038/s41598-022-06084-z

**Published:** 2022-02-07

**Authors:** Isaku Okamoto, Kiyoaki Tsukahara, Hiroki Sato

**Affiliations:** Department of Otorhinolaryngology, Head and Neck Surgery, Tokyo Medical University, 6-7-1 Nishishinjuku, Shinjuku-ku, Tokyo, 160-0023 Japan

**Keywords:** Head and neck cancer, Cancer immunotherapy

## Abstract

Nivolumab, an immune checkpoint inhibitor, is beneficial to patients with platinum-refractory recurrent or metastatic head and neck squamous cell carcinoma (R/M-HNSCC). However, platinum-sensitive R/M-HNSCC has not yet been studied. Hence, in this prospective study, we evaluated the efficacy and safety of nivolumab in patients with platinum-sensitive R/M-HNSCC. This prospective single-arm study was conducted in a single institution in Japan. Patients with platinum-sensitive R/M-HNSCC (defined as head and neck cancer that recurred or metastasized at least 6 months after platinum-based chemotherapy or chemoradiotherapy) were enrolled. The primary endpoint was overall survival (OS). The secondary endpoints were progression-free survival (PFS), overall response rate (ORR), immune-related adverse events (irAEs), and quality of life (QOL). This study was registered at the University Hospital Medical Information Network Clinical Trials Registry (UMIN000031324). Twenty-two patients with platinum-sensitive R/M-HNSCC were enrolled. The median OS was 17.4 months, and the 1-year OS rate was 73%. The median PFS was 9.6 months, 1-year PFS rate was 48%, and ORR was 36%. Sixteen irAEs were recorded in 12 patients; however, no grade 4 or 5 irAEs were observed. The QOL assessments revealed that nivolumab did not decrease the QOL of patients. Nivolumab is effective against platinum-sensitive R/M-HNSCC with acceptable safety.

## Introduction

The prognosis of patients with recurrent or metastatic head and neck squamous cell carcinoma (R/M-HNSCC) is poor, with a median overall survival (OS) of less than 1 year^1–3^. In 2016, an international phase III trial (CheckMate-141) revealed that, compared with the standard-of-care, nivolumab (an immune checkpoint inhibitor) prolonged the OS of patients with platinum-refractory R/M-HNSCC. In the CheckMate-141 study, treatment with nivolumab led to a median OS of 7.5 months, median progression-free survival (PFS) of 2.04 months, and an overall response rate (ORR) of 13.3%^4^. Thus, the National Comprehensive Cancer Network (NCCN) Guidelines recommend nivolumab along with the EXTREME regimen as a category 1 treatment for patients with R/M-HNSCC with a history of platinum therapy^5,6^. Several studies, including a real-world retrospective study, have reported the efficacy of nivolumab, with median OS ranging from 8.7 to 13.0 months, median PFS ranging from 1.9 to 3.7 months, and ORR ranging from 13 to 30%^7–10^.

The concept of platinum refractoriness initially used for treatment strategies for recurrent ovarian cancer^11,12^ has recently been applied to R/M-HNSCC^13^. Tumors are considered platinum-refractory if they recur or metastasize within 6 months after platinum-based chemotherapy or chemoradiotherapy. In contrast, tumors are considered platinum-sensitive if they recur or metastasize more than 6 months after platinum-based chemotherapy or chemoradiotherapy. Notably, CheckMate-141 only enrolled patients with platinum-refractory R/M-HNSCC, and no prospective study has been conducted on the efficacy and safety of nivolumab for platinum-sensitive R/M-HNSCC. The KEYNOTE 048 trial suggested that pembrolizumab, a programmed cell death protein 1 (PD-1) inhibitor, is effective against platinum-sensitive R/M-HNSCC^14^; pembrolizumab is also considered a category 1 therapy in the NCCN Guidelines^5^. Nivolumab, similar to pembrolizumab, is a PD-1 inhibitor, and it may be effective against platinum-sensitive R/M-HNSCC. The aim of this study was to prospectively evaluate the efficacy and safety of nivolumab in patients with platinum-sensitive R/M-HNSCC.

## Results

### Patient characteristics

From February 16, 2018, to December 31, 2019, 61 patients with recurrent or metastatic head and neck cancer received at least one cycle of nivolumab. Among these, 39 patients were excluded owing to platinum resistance (*N* = 35), non-squamous cell carcinoma (*N* = 3), and age > 75 years (*N* = 1). Twenty-two patients who met the inclusion criteria were enrolled in this study. The data cutoff date was December 31, 2020. Detailed data of all patients are provided in the Supplementary Information.

Table 1 lists the age, sex, smoking history, drinking history, Eastern Cooperative Oncology Group performance status (ECOG PS), tumor-node-metastasis (TNM) classification and staging at recurrence or metastasis, and programmed cell death-ligand 1 (PD-L1) expression. The median age of patients was 63 (range, 42–74) years. The primary tumor sites included the nasopharynx (*N* = 6) and maxillary sinus (*N* = 4). PD-L1 expression was < 1% in 5 patients, ≥ 1% in 16 patients, ≥ 20% in 12 patients, ≥ 40% in 8 patients, and unmeasured in 1 patient. Treatment modalities prior to nivolumab administration are presented in Table 2. Previous exposure to platinum included induction chemotherapy (docetaxel/cisplatin/5-fluorouracil, *N* = 3), definitive concurrent chemoradiotherapy (*N* = 13), postoperative chemoradiotherapy (*N* = 6), and systemic therapy for recurrence or metastasis (cetuximab/cisplatin/5-fluorouracil, *N* = 1). In addition, one patient underwent a duplicate platinum-containing regimen. Cetuximab was used prior to nivolumab treatment as a definitive concurrent radiotherapy (*N* = 1) and as a systemic therapy for countering the recurrence or metastasis of disease (cetuximab/paclitaxel, *N* = 3; cetuximab/cisplatin/5-fluorouracil, *N* = 1). The median period between the date of the last administration of platinum and the date of disease progression was 351 (range, 201–1,081) days, whereas the median period between the date of the last administration of platinum and the date of the first administration of nivolumab was 397 (range, 211–1,110) days. The median number of cycles of nivolumab administration was 9 (range, 1–57).

**Table 1:** Patients’ characteristics.

Patient’s characteristic	No. of patients	%
**Age, years**		
Mean	61	
Median	63	
Range	42–74	
**Sex**		
Male	20	90.9
Female	2	9.1
**Smoking**		
Never	5	22.7
Ever	17	77.3
**Alcohol consumption**		
Never	7	31.8
Ever	15	68.2
**ECOG performance status**		
PS 0	18	81.8
PS 1	4	18.2
**Primary tumor site**		
Nasopharynx	6	27.3
Oropharynx	5	22.7
p16 positive	4	18.2
p16 negative	0	0
p16 unknown	1	4.5
Hypopharynx	6	27.3
Larynx	1	4.5
Maxillary sinus	4	18.2
**T category**		
TX	15	68.2
T1/T2/T3	0	0
T4	7	31.8
**N category**		
N0	17	77.3
N1/N2/N3	5	22.7
**M category**		
M0	10	45.5
M1	12	54.5
**UICC Stage**		
Stage I/II/III	0	0
Stage IV	22	100
**Reason for unresectability**		
Locally advanced	3	13.6
Recurrent	7	31.8
Metastatic	12	54.5
**PD-L1 expression**		
< 1%	5	22.7
≥ 1%	16	72.7
≥ 20%	12	54.5
≥ 40%	8	36.4
Unknown	1	4.5

**Table 2:** Treatment profiles.

Treatment	No. of patients	%
**Previous treatment**		
Surgery	10	45.5
Radiation therapy	22	100.0
Chemotherapy	22	100.0
**Previous platinum-containing regimen**		
Induction chemotherapy with docetaxel + cisplatin + 5-fluorouracil	3	13.6
Concurrent chemoradiotherapy with cisplatin	13	59.1
Postoperative chemoradiotherapy with cisplatin	6	27.3
Cetuximab + cisplatin + 5-fluorouracil for unresectable disease	1	4.5
Previous cetuximab-containing regimen	5	22.7
Concurrent radiotherapy with cetuximab	1	4.5
Cetuximab + cisplatin + 5-fluorouracil for unresectable disease	1	4.5
Cetuximab + paclitaxel for unresectable disease	3	13.6
**Duration from the last platinum administration – days, median (range)**		
To the confirmation of recurrence	351 (201–1081)	
To the first administration of nivolumab	397 (211–1110)	
**Number of previous regimens for unresectable disease**		
1	18	81.8
2	4	18.2
Number of nivolumab administration–median (range)	9 (1–57)	
**Reason for nivolumab discontinuation**		
Disease progression	7	31.8
Adverse event	4	18.2
Patient's choice	1	4.5
Physician's choice	1	4.5

### Efficacy

**Figure 1:**
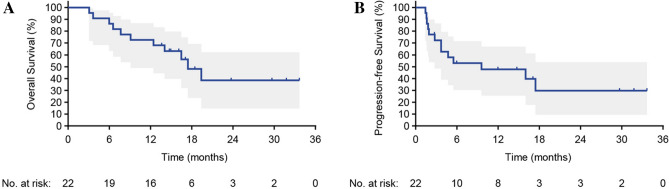
Kaplan–Meier curves of overall survival and progression-free survival. Kaplan–Meier curves of (**A**) overall survival and (**B**) progression-free survival. Vertical lines show censored events, and the shaded region represents a 95% confidence interval.

**Figure 2:**
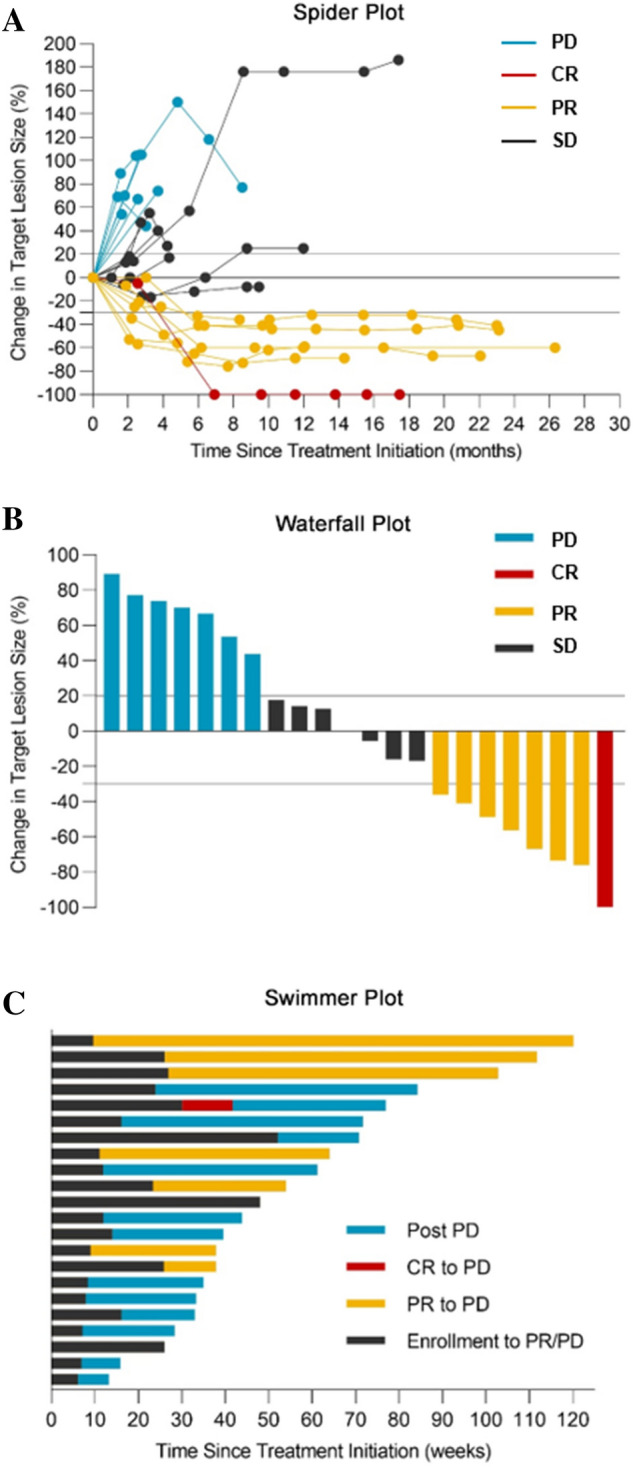
Responses of patients with platinum-sensitive R/M-HNSCC to nivolumab based on the RECIST guidelines (version 1.1). (**A**) Change in the sum of target lesions over time from baseline to PD. The upper dashed line represents the threshold for PD, and the lower dashed line shows the threshold for PR. (**B**) Best reduction in the target lesions from baseline. The upper dashed line represents the threshold for PD (20% increase in the sum of the longest diameter of the target lesions). The lower dashed line shows the threshold for PR (30% decrease in the sum of the longest diameter of the target lesions). (**C**) Time to response and duration of survival. Each bar represents a single patient, with the length of the bar corresponding to overall survival.

After data cutoff, the median follow-up period for all patients was 14.9 (range, 3.1–33.7) months. The median OS was 17.4 months (95% confidence interval [95% CI], 9.1 months to not calculable [N/C]), and the 1-year OS rate was 73% (95% CI, 49%–87%) (Fig. 1A). The median PFS was 9.6 months (95% CI, 2.8 months to N/C), and the 1-year PFS rate was 48% (95% CI, 26%–67%) (Fig. 1B). The responses were as follows: complete response (CR; one patient), partial response (PR; seven patients), stable disease (SD; seven patients), and progressive disease (PD; seven patients). The ORR was 36% and the disease control rate (DCR) was 68%. Tumor shrinkage was observed in 11 patients (50%) (Fig. 2A,B). One patient, in whom the target lesions were assessed as CR, was evaluated as PD owing to the appearance of new distant metastatic lesions during the follow-up (Fig. 2C). Table 3 shows the OS, PFS, and response rates by primary site; HPV/p16 status; and local progression, recurrence, or metastasis status. The survival and response rates of the non-nasopharyngeal cancer group and the CheckMate-141 target and non-CheckMate-141 target groups are also shown.

**Table 3:** Efficacy by primary tumor site.

Primary tumor site	ALL patients (n = 22)		Median OS (95% CI)	1-year OS rate (95% CI)	Median PFS (95% CI)	1-year PFS rate (95% CI)	ORR	DCR
	No	%	(months)	(%)	(months)	(%)	(%)	(%)
Nasopharynx	6	27.3	16.5 (3.1–N/C)	66.7 (19.5–90.4)	4.7 (1.4–N/C)	50.0 (11.1–80.4)	33.3	66.7
Oropharynx	5	22.7	17.4 (6.0–N/C)	60.0 (12.6–88.2)	5.5 (1.64–N/C)	40.0 (5.2–75.3)	40.0	80.0
p16 positive	4	18.2	17.4 (5.97–N/C)	75.0 (12.8–96.1)	5.48 (3.7–N/C)	50.0 (5.8–84.5)	25.0	75.0
p16 negative/unknown	1	4.5	6.5 (N/C – N/C)	N/C (N/C)	1.6 (N/C – N/C)	N/C (N/C)	100.0	100.0
Hypopharynx	6	27.3	N/C (7.7–N/C)	83.3 (27.3–97.5)	9.6 (1.8–N/C)	41.7 (5.6–76.7)	50.0	83.3
Larynx	1	4.5	3.6 (N/C – N/C)	N/C (N/C)	1.6 (N/C – N/C)	N/C (N/C)	0.0	0.0
Maxillary sinus	4	18.2	N/C (N/C – N/C)	N/C (12.4–N/C)	N/C (1.94 – N/C)	75.0 (12.8–96.1)	50.0	50.0
non-Nasopharynx	16	72.7	19.4 (7.7–N/C)	75.0 (46.3–89.8)	9.6 (1.9–N/C)	46.8 (20.9–69.2)	43.8	68.8
Oropharynx, Hypopharynx, Larynx	12	54.5	17.4 (5.97–N/C)	66.7 (33.7–0.86.0)	5.5 (1.6–N/C)	35.2 (9.6–62.8)	41.7	75.0
Nasopharynx, Maxillary sinus	10	45.5	N/C (3.1–N/C)	80.0 (40.9–94.6)	N/C (1.4–N/C)	60.0 (25.3–82.7)	40.0	60.0
Local advanced	3	13.6	N/C (9.1–N/C)	66.7 (5.4–94.5)	N/C (4.7–N/C)	66.7 (5.4–94.5)	66.7	66.7
Recurrent	7	31.8	14.1 (3.1–N/C)	57.1 (17.2–83.7)	N/C (1.4–N/C)	53.6 (13.2–82.5)	0.0	0.0
Metastasis	12	54.5	19.4 (7.7–N/C)	83.3 (48.2–95.6)	7.5 (1.8–N/C)	41.7 (15.2–66.5)	33.3	66.7

### Safety

Table 4 lists the immune-related adverse events (irAEs) in all patients administered nivolumab. In total, 16 irAEs of different grades were detected in 12 patients: liver dysfunction (*N* = 5), interstitial lung disease (*N* = 4), hypothyroidism (*N* = 4), hyperthyroidism (*N* = 1), and dermatitis (*N* = 2). The following grade 3 or higher irAEs occurred in four patients: liver dysfunction (*N* = 3) and hypothyroidism (*N* = 1).

**Table 4:** Immune-related adverse events.

Patients, No. (%)	All grades	Grade 1	Grade 2	Grade 3	Grade 4	Grade 5
Dermatitis	2 (4.5)	2 (9.1)	0	0	0	0
Interstitial lung disease	4 (18.2)	3 (13.6)	1 (4.5)	0	0	0
Hypothyroidism	4 (18.2)	2 (9.1)	1 (4.5)	1 (4.5)	0	0
Hyperthyroidism	1 (4.5)	0	1 (4.5)	0	0	0
Liver dysfunction	5 (22.7)	0	2 (9.1)	3 (13.6)	0	0

### Quality of life assessments

**Figure 3:**
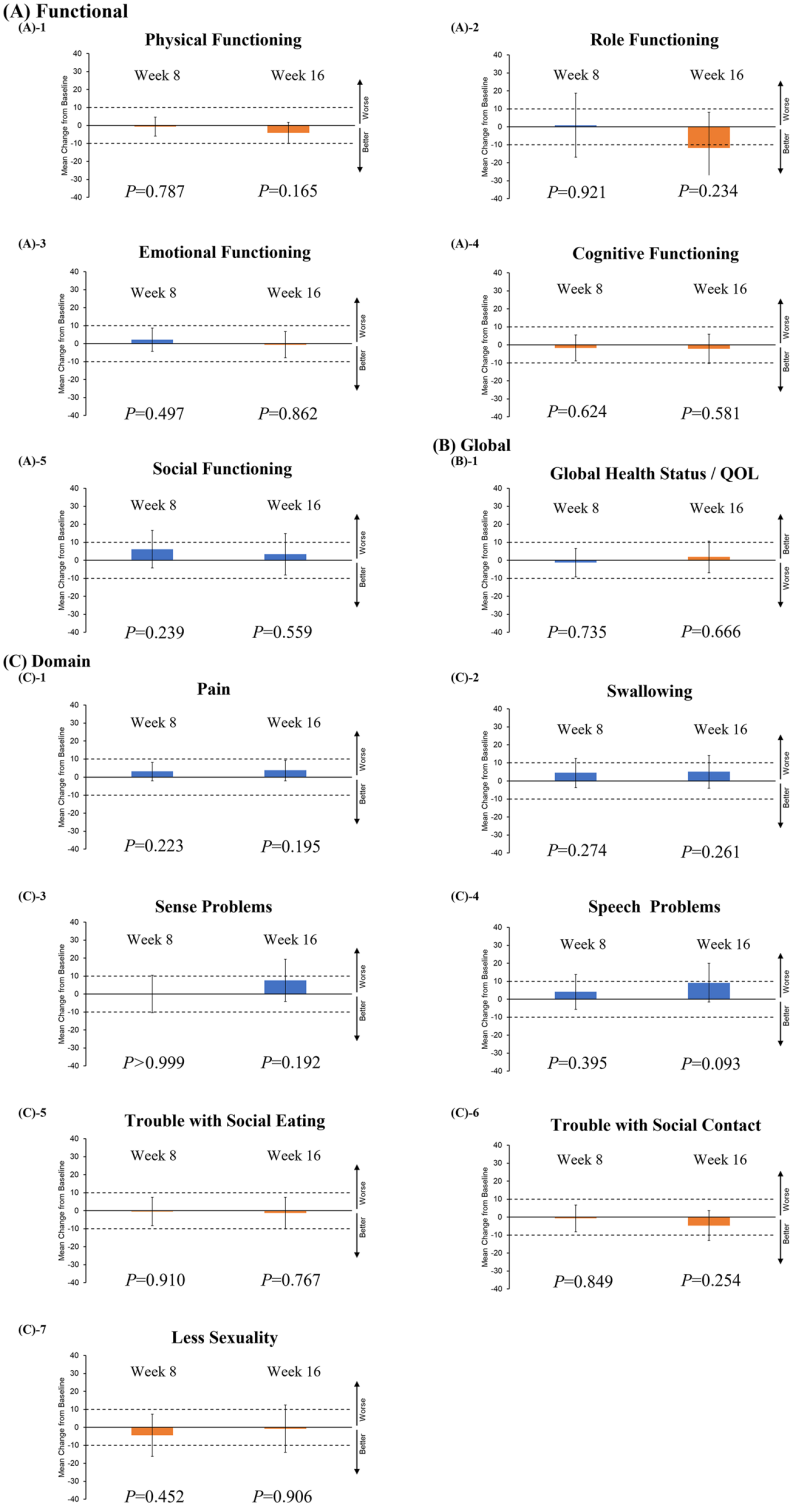
Quality of life assessments. (**A**) Functional scales (physical, role, emotional, cognitive, and social activities) and (**B**) global health status were assessed using the EORTC QLQ-C30. (**C**) Domain scales (pain, swallowing, sense problems, speech problems, trouble with social eating, trouble with social contact, and reduced sexuality) were assessed using the QLQ-H&N35. All scales ranged from 0 to 100, and score changes of at least 10 points (dashed line) were considered clinically significant. Higher values for functional and domain scales indicate poor functioning, whereas higher values for global health status indicate better functioning. The I bar indicates a 95% confidence interval.

Functional scales (physical, role, emotional, cognitive, and social functioning) and global health status were assessed using the European Organization for Research and Treatment of Cancer (EORTC) Quality of Life Questionnaire (QLQ) Core 30 Module (QLQ-C30). Domain scales (pain, swallowing, sense problems, speech problems, trouble with social eating, trouble with social contact, and reduced sexuality) were assessed using the EORTC QLQ Head and Neck Cancer Module (QLQ-H&N35). Detailed data regarding the quality of life (QOL) scores are provided in the Supplementary Information. The data of 19 patients, excluding the 3 patients who died by week 8, were analyzed. Figure 3 shows the QOL scores relative to the baseline value at 8 and 16 weeks after the initiation of nivolumab. There was no significant change in the QOL of patients treated with nivolumab.

## Discussion

The aim of this study was to determine the efficacy and safety of nivolumab in patients with platinum-sensitive R/M-HNSCC through a prospective study. The main outcomes of this study were the median OS of 17.4 months, median PFS of 9.6 months, ORR of 36%, and DCR of 68%. Although the number of patients in the study by primary site was too small to ensure the certainty of the results, there was no clear effect of the primary site on efficacy.

Considering that the prognosis of patients with R/M-HNSCC is poor at less than 1 year^1–3^, it is evident that treatment with nivolumab improved the OS. To the best of our knowledge, this is the first prospective study to demonstrate the efficacy of nivolumab in patients with platinum-sensitive R/M-HNSCC only.

No prospective study has examined the effect of nivolumab in patients with platinum-sensitive head and neck cancer or with other carcinomas. The concept of platinum-resistant and platinum-sensitive cancer was originally used in the treatment of recurrent ovarian cancer and has recently been applied to R/M-HNSCC^11,12^. In recurrent ovarian cancer, patients with platinum-sensitive cancer are more likely to respond to subsequent chemotherapy and have a better prognosis than those with platinum-resistant cancer^11,12^. For R/M-HNSCC, the effects of the EXTREME regimen on the OS of two groups of patients with R/M-HNSCC, namely patients with platinum-resistant and platinum-sensitive R/M-HNSCC, have been compared^6,15,16^. Sato et al. reported significantly better OS in patients with platinum-sensitive R/M-HNSCC than in those with platinum-resistant R/M-HNSCC (median OS: 19.9 vs. 10.6 months; *P* = 0.02)^15^. In contrast, Sano et al. showed that the OS of the non-platinum-refractory group was significantly higher than that of the platinum-refractory group (median OS: 7 vs. 13 months; *P* = 0.018)^16^. Hori et al. reported significantly better PFS in the non-platinum-refractory group than in the platinum-refractory group of nivolumab-treated patients with R/M-HNSCC (median PFS: 13 vs. 38 weeks; *P* = 0.006)^17^. Although the results of Hori et al. cannot be directly compared with those of CheckMate-141 for platinum-refractory R/M-HNSCC or with those of retrospective studies, they provide further evidence of the benefits of nivolumab in patients with R/M-HNSCC^4,7–10,17^. Similar to patients with recurrent ovarian cancer, those with platinum-sensitive R/M-HNSCC may respond well to subsequent pharmacotherapy. In Japan, in addition to nivolumab, pembrolizumab is indicated for R/M-HNSCC. In 2019, Burtness et al. studied the efficacy of pembrolizumab as a primary treatment in patients with R/M-HNSCC without prior chemotherapy in the international phase III KEYNOTE-048 trial^14^. Among patients with PD-L1 combined positive scores ≥ 20, those treated with pembrolizumab alone exhibited significantly higher OS than the group administered the EXTREME regimen (cetuximab with chemotherapy group) (median: 14.9 vs. 10.7 months, HR 0.61 [95% CI, 0.45–0.83], *P* = 0.0007). The NCCN Guidelines also recommend pembrolizumab as a category 1 treatment^5^. However, the KEYNOTE-048 trial included patients with R/M-HNSCC who underwent chemotherapy-free treatments; hence, the correlation between platinum sensitivity and pembrolizumab treatment outcomes is unknown. To determine the efficacy of pembrolizumab for platinum-refractory R/M-HNSCC, Cohen et al. conducted KEYNOTE-040^18^. The median OS was 8.4 (95% CI, 6.4–9.4) months with pembrolizumab and 6.9 (95% CI, 5.9–8.0) months with the physician’s choice of therapy (HR 0.80, 0.65–0.98; *P* = 0.0161), indicating an improvement in OS but still a poor outcome. Additional studies are warranted to compare outcomes between nivolumab and pembrolizumab as a first-line treatment for platinum-sensitive R/M-HNSCC.

Suzuki et al. reported a reduced tumor growth rate after nivolumab treatment and found that the size of the target lesions affected OS and PFS as measured by the sum of their diameter^19^. In our institution, we followed up with the patients every month after completing the local treatment for head and neck cancer and performed imaging studies every 3–4 months. In patients with suspected recurrence, imaging was performed at relatively short intervals. In this study, tumor burden and growth rate were not measured. However, R/M-HNSCC was likely diagnosed at a relatively early stage, resulting in the immediate administration of nivolumab. We believe this is one of the reasons for the favorable OS and PFS observed in this study. Unlike multicenter studies, our study did not require a specific time for the enrollment of patients, which enabled the rapid initiation of treatment, an advantage of single-arm studies conducted in a single institution.

In a sub-analysis of data of CheckMate-141, Ferris et al. compared the OS of patients with and without prior exposure to cetuximab^20^. They reported that patients without prior exposure were at a lower risk of death from nivolumab use. Cetuximab binds to the epidermal growth factor receptor on tumor cells but promotes the growth of immunosuppressive regulatory T cells in the tumor microenvironment^21^. Patients whose lesions worsen after cetuximab treatment are less likely to respond to immunotherapy owing to the proliferation of immunosuppressive regulatory T cells, such as regulatory T cells and myeloid-derived suppressor cells^21,22^. In our study, five patients (22.7%) with a history of cetuximab treatment before nivolumab treatment could not attain an endpoint for previous cetuximab use. Therefore, it is not known whether previous cetuximab use had any effect on patient response. The CheckMate-141 QOL assessments showed that nivolumab does not reduce the QOL (compared with other chemotherapy or molecularly targeted drug treatments)^4,23^. This observation is not limited to R/M-HNSCC; it has also been reported for melanoma, non-small cell lung cancer, and renal cell carcinoma^24–26^. Our results also showed no reduction in the QOL of patients after treatment with nivolumab.

Among the 22 patients, 16 irAEs occurred in 12 patients. Grade 3 or higher irAEs were liver dysfunction (N = 3) and hypothyroidism (N = 1). Nivolumab was discontinued due to irAEs in three patients with grade 3 liver dysfunction. After treatment with steroids, all patients showed an improvement in liver dysfunction. There were three other patients who discontinued nivolumab due to the deterioration of their general condition. Safety was not a significant problem.

There were some limitations to the study. This was a single-arm study, and although the enrollment number was set at 50 patients, it was not reached. Sixty-one patients with R/M-HNSCC received nivolumab within the target period, but 35 were excluded due to platinum resistance. The effects of nivolumab in patients with platinum-resistant and platinum-sensitive R/M-HNSCC should be further examined in a multicenter prospective study with a higher number of patients. Therefore, as the next step of the study, we are investigating the results of treatment in platinum-sensitive and platinum-resistant groups in a multicentered study. The results of this study will be published separately. In addition, the outcomes of nivolumab and pembrolizumab as first-line treatments for patients with platinum-sensitive R/M-HNSCC with a history of platinum use should be compared in future studies. These studies are necessary to establish more effective treatment strategies for patients with R/M-HNSCC.

In conclusion, our results suggest that nivolumab is highly effective in treating platinum-sensitive R/M-HNSCC, with no reduction in the QOL following treatment. These results may have implications for the current treatment regimen for R/M-HNSCC.

## Patients and methods

### Study design

This was a single-institution, open-label, single-arm, phase II interventional study conducted in Japan to evaluate the efficacy of nivolumab in patients with R/M-HNSCC. The study was approved by the ethics committee of Tokyo Medical University (SH3946) and registered at the University Hospital Medical Information Network Clinical Trials Registry prior to the recruitment of patients (UMIN000031324; date of first registration;15/02/2018). The study adhered to the tenets of the Declaration of Helsinki, and informed consent was obtained from all the patients.

### Patients

Patients with R/M-HNSCC were screened for eligibility for this study. The number of patients expected to be enrolled was 50, which was calculated based on the number of patients with R/M-HNSCC treated in our institution (approximately 60 patients per year), the expected rate of enrollment (40%), and the study period to enroll patients (2 years). The main exclusion criteria were prior exposure to nivolumab; platinum refractoriness, defined as disease progression within 6 months after the last administration of platinum; non-squamous cell carcinoma; and age over 75 years. The complete inclusion and exclusion criteria are provided in the Supplementary Protocol.

### Outcomes and assessments

The primary endpoint was OS. The period of OS was defined as the duration between the date of nivolumab initiation and the date of the last follow-up or the patient’s death, whichever occurred first. The secondary endpoints were PFS, ORR, and QOL. The period of PFS was defined as the duration between the date of nivolumab initiation and the date of objective disease progression or patient’s death from any cause, whichever occurred first. Tumor response was assessed by three radiologists in our institution according to the Response Evaluation Criteria in Solid Tumors Guideline (version 1.1)^27^. The QOL was assessed using the EORTC QLQ-C30, a basic quality of life questionnaire used for patients with malignancies, and the EORTC QLQ-H&N35, a disease-specific questionnaire^28,29^. The EORTC QLQ-C30 and QLQ-H&N35 were translated into Japanese and answered by the patients prior to treatment initiation and at 8 and 16 weeks after treatment initiation. These scores ranged from 0 to 100, with higher scores indicating higher functioning and symptom burden, except for the global health status items. Higher scores indicate lower functioning and symptom burden. TNM was classified using the Union for International Cancer Control stage, 7^th^ edition^30^. The discontinuation criteria for nivolumab were specified in the protocol based on the grade of irAEs, but no weight loss criterion was set^4^. The irAEs were assessed using the Common Terminology Criteria for Adverse Events version 4.0^31^.

### Administration of nivolumab and PD-L1 measurement

Nivolumab was administered as a single intravenous dose (3 mg/kg body weight) every 2 weeks. A single treatment cycle lasted 2 weeks, and imaging assessment was carried out every 4–8 cycles. Treatment was continued until the confirmation of objective disease progression, the occurrence of unacceptable toxicity, or other reasons assessed by the attending physician. However, even after confirming progression based on clinical or imaging findings, treatment was continued when the attending physician thought that clinical benefit was likely^32^.

PD-L1 was measured in CheckMate-141, and Dako 28–8 antibody (Dako, Carpinteria, CA) was used for immunohistochemical assay. The scoring systems include the Tumor Proportion Score (TPS), which measures PD-L1 on tumor cells, and the Combined Positive Score (CPS), which measures PD-L1 expressed on not only tumor cells but also macrophages and lymphocytes. In this study, the measurement was performed using the TPS, and not the CPS^32^.

### Statistical analysis

OS and PFS were estimated using the Kaplan–Meier method. QOL assessments were conducted using a repeated measures linear mixed model, with repeated measures covariance structure as a composite symmetry, each QOL score as the dependent variable, time of measurement as a fixed factor, and subjects as a variable. In this case, the least squares mean and the 95% CI at each measurement point were calculated. The estimated mean and 95% CI were also calculated for changes based on the pre-nivolumab scores (baseline), and the significant change from the pre-nivolumab scores was tested. No correction for multiplicity was used. Statistical analysis for OS and PFS was performed using EZR, a statistical software that extends the functions of R and R commander; it is available on the Jichi Medical University Saitama Medical Center website^33^. SPSS statistics version 22.0 (IBM Japan, Ltd., Tokyo, Japan) was used for the statistical analysis of QOL. Results with *P* < 0.05 were considered statistically significant.

## Supplementary Information

Supplementary Information.

## Data Availability

The datasets generated in the current study are available from the corresponding author on request.
