# Supplementary material for: Single-center prospective study on the efficacy of nivolumab against platinum-sensitive recurrent or metastatic head and neck squamous cell carcinoma
Source: Sci Rep. 2022 Feb 7;12:2025. doi: 10.1038/s41598-022-06084-z (PMC8821556; doi:10.1038/s41598-022-06084-z)
Supplement: Supplementary file 1 — Supplementary Information. [file 41598_2022_6084_MOESM1_ESM.doc]

**Supplementary** **Information**

**Study Protocol**

**A single-center prospective study on the efficacy of nivolumab against platinum-sensitive recurrent or metastatic head and neck squamous cell carcinoma**

1st edition

Date of completion: 31 October 2017

1. Background

A combination of surgery, radiation, and chemotherapy is the preferred treatment for advanced head and neck cancer. Patients with unresectable recurrent or metastatic squamous cell carcinoma of the head and neck (R/M-SCCHN) have a poor prognosis and usually have a life expectancy of approximately one year. FP (5FU, CDDP) is the recommended treatment for this condition, but its poor efficacy and toxicity have made long-term treatment difficult. In the EXTREME study, cetuximab was combined with cisplatin or carboplatin and FP (FPE). A high response rate of 36% was observed for the combination compared to 20% for FP alone. In the case of FPE, up to 6 cycles are provided, but in some cases, only 2 or 3 cycles are administered in practice due to adverse events. After that, cetuximab can be administered weekly until progressive disease (PD) or until the manifestation of unacceptable adverse events. Thus, instances of long-term survival of more than two to three years, which was previously unthinkable, have increased, and a regimen that includes cetuximab has become the current standard of care.

On March 24, 2017, the use of nivolumab, an immune checkpoint inhibitor for patients with recurrent or metastatic head and neck cancer, was approved for insurance coverage in Japan. A few drugs are available to treat recurrent or metastatic head and neck cancers, and there are no established treatments for patients suffering from this condition; therefore, this drug is expected to serve as a new treatment option. The international Phase III trial (Checkmate-141 study) of nivolumab revealed significant OS prolongation in patients with platinum-resistant recurrent or metastatic head and neck cancer over the control arm of anticancer drugs and cetuximab. As mentioned above, FPE is the standard of care for recurrent and metastatic head and neck cancer. Still, it can further prolong survival and improve the quality of life for patients with poor prognosis. However, because the Checkmate 141 study included patients whose disease progressed within 6 months of completion of platinum-based chemotherapy, there is insufficient evidence in patients whose disease progressed after 6 months of completion of platinum-based chemotherapy.

2. Objectives

The purpose of this study is to evaluate the effects of nivolumab in patients with relapse or metastases 6 months after the completion of platinum-based chemotherapy.

3. Eligible patients

Expected number of patients: 50

(1) Inclusion criteria

Patients who met all the following criteria were included in this study:

1) Patients manifesting R/M-SCCHN six months after completing platinum-based chemotherapy

2) Primary site: the maxillary sinus, oral cavity, nasopharynx, oropharynx, hypopharynx, or larynx

3) Age 20-75 years

4) Eastern Cooperative Oncology Group performance-status (ECOG PS) score 0-1

5) Adequate organ functions defined as indicated below before registration in 14 days

(1) White Blood Cell ≧ 3,500 /mm^3^, White Blood Cell ≦ 12,000 /mm^3^

(2) neutrophil ≧ 2,000 /mm^3^

(3) Platelet ≧ 100,000 /mm^3^

(4) Hemoglobin ≧ 9.0 g/ dL

(5) Total bilirubin ≦ 1.5 mg/dL

(6) aspartate aminotransferase, alanine aminotransferase ≧ 100 IU/L

(7) Creatinine < 1.2 mg/dL

6) Written informed consent

(2) Exclusion criteria

Patients who met any of the following conditions were excluded from this study:

1) Patients with a history of severe drug allergy of nivolumab and similar drugs.

2) Patients with symptomatic brain metastasis and meningeal metastasis.

3) Histologically proven primary unknown squamous cell carcinoma, salivary gland cancer, non-squamous cell cancer.

4) Patients with symptomatic autoimmune disease or history of autoimmune disease.

5) Patients with infection.

6) Patients with gastrointestinal disease (intestinal paralysis, bowel obstruction)

7) Patients with interstitial pneumonia or pulmonary fibrosis.

8) Uncontrolled diabetes.

9) Uncontrolled heart disorder.

10) Sever liver damage.

11) Patients with diarrhea.

12) Pregnant or lactating women or women with childbearing potential.

13) Men expecting partner's pregnancy.

14) Inadequate physical condition, as diagnosed by the attending physician

(3) Evidence for deciding the number of cases

This study is a single-arm phase II trial. The rate of recurrent or metastatic head and neck cancers at our institution is approximately 4 cases per month. Only 30% to 40% of the patients met the selection criteria and were eligible for enrollment. The study enrollment period is set at 2 years, and it is estimated that we will be able to enroll nearly 50 patients.

4. Material and Method

(1) Type and design

This is a prospective, interventional study in which nivolumab will be administered to subjects to evaluate treatment effects. It is an invasive study due to drug administration.

(2) Method

1) Patient enrollment

Patient enrollment will be determined by the principal investigator and sub-investigators.

2) Enrollment procedures

Confirm that eligible patients meet all eligibility criteria and none of the exclusion criteria, complete the Enrollment Eligibility Confirmation Form, and deliver it to the principal investigator. The principal investigator will review the information presented in the Enrollment Eligibility Confirmation Form and issue a registration number.

3) Treatment plan and treatment change criteria

Within 4 weeks of enrollment completion, treatment as per the protocol will be initiated under the direction of the principal investigator.

4) Protocol treatment

Nivolumab is administered at a single dose of 3 mg/kg (body weight) IV at 2-week intervals, which continues from the protocol initiation date until treatment is discontinued due to disease progression, unacceptable toxicity, other reasons.

5) Nivolumab discontinuation criteria

Discontinue nivolumab in the event of an adverse event (e.g., shock, anaphylactoid symptoms, etc.) determined to be attributable to this drug or in the event of a patient refusal.

6) Withdrawal criteria and resumption criteria

At the initiation of nivolumab treatment (after the pre-course withdrawal period) or at any time during treatment, treatment should be continued after confirming that various laboratory tests have met the dosage criteria. If the dosage criteria are not met, the drug should be withdrawn and resumed according to the following criteria.

7) Criteria for drug withdrawal

If any of the following withdrawal criteria are met during treatment, treatment should be discontinued immediately, and treatment should be resumed while waiting for the patient's recovery to reach or exceed the withdrawal criteria.

In cases where the baseline is close to the withdrawal criteria, the investigator/assigned physician will decide on whether to discontinue treatment at the time the withdrawal criteria are violated.

| Inspection item | | Criteria for drug withdrawal | |
| --- | --- | --- | --- |
| Criteria for drug withdrawalBlood toxicity | White blood cell | < | 3,000 /mm^3^ |
|  | Neutrophil | < | 1,500 /mm^3^ |
|  | Platelet | < | 100,000 /mm^3^ |
|  | Hemoglobin | < | 9.0 g/ dL |
| Non-blood toxicity | Total bilirubin | ≧ | 2.0 mg/dL |
|  | Aspartate aminotransferase | ≧ | 100 IU/L |
|  | Alanine aminotransferase | ≧ | 100 IU/L |
|  | Creatinine | ≧ | 1.2 mg/dL |
|  | Other adverse events | ≧ | Grade2 |

8) Criteria for resuming administration

In the case of nivolumab administration, the following "Resumption Criteria" should be met before initiating nivolumab administration. The dose at the resumption of nivolumab administration is determined after confirming that the dose reduction criteria have been met.

If the withdrawal period for adverse events exceeds 28 days, the treatment is discontinued in this patient.

| Inspection item | | Criteria for resuming administration | |
| --- | --- | --- | --- |
| Blood toxicity | White blood cells | ≧ | 3,000 /mm^3^ |
|  | Neutrophil | ≧ | 1,500 /mm^3^ |
|  | Platelet | ≧ | 100,000 /mm^3^ |
|  | Hemoglobin | ≧ | 9.0 g/ dL |
| Non-blood toxicity | Total bilirubin | < | 2.0 mg/dL |
|  | Aspartate aminotransferase | < | 100 IU/L |
|  | Alanine aminotransferase | < | 100 IU/L |
|  | Creatinine | < | 1.2 mg/dL |
|  | Other adverse events | < | Grade1 |

9) Drug information

The following is a summary of the drug used in this study. Please refer to the most recent package insert for the drug for further information.

Nivolumab:

Generic name: Nivolumab

Product Name: Opdivo

[Contraindication]

Patients with a history of hypersensitivity to any of the ingredients in this product

[Careful with doses]

1) Patients with concomitant autoimmune disease or a history of chronic or recurrent autoimmune disease [autoimmune disease may be aggravated].

2) Patients with interstitial lung disease or a history of interstitial lung disease [there is a risk of worsening of interstitial lung disease].

Serious side effects.

1) Interstitial pulmonary disease.

Since interstitial lung disease (3.6%) such as pulmonary inflammation, pulmonary infiltration, and pulmonary disorder may occur, clinical symptoms such as cough, dyspnea, fever, and abnormal lung sounds (hair twirling sound) should be carefully monitored, and if any abnormality is observed, chest X-ray, chest CT, and serum markers, etc. should be conducted promptly. If interstitial lung disease is suspected, the administration should be discontinued, and appropriate measures such as the administration of corticosteroids should be taken.

2) Myasthenia gravis, myocarditis, myositis, and rhabdomyolysis

Myasthenia gravis (frequency unknown*), myocarditis (frequency unknown*), myositis (0.1%), and rhabdomyolysis (frequency unknown*) may occur, and some cases have been further complicated by these conditions. Muscle weakness, ptosis, dyspnea, dysphagia, elevated CK (CPK), ECG abnormality, and elevated blood and urinary myoglobin should be carefully monitored. If any abnormality is observed, the administration should be discontinued, and appropriate measures such as the administration of corticosteroids should be taken. As respiratory failure may progress rapidly due to myasthenia gravis, precautions should be taken to ensure that the patient's respiratory condition does not worsen; hepatic dysfunction and jaundice with increases in AST (GOT), ALT (GPT), and γ-GTP may occur, and if any abnormalities are observed, the patient should be carefully monitored. The administration should be discontinued, and appropriate measures taken.

3) Colitis, severe diarrhea

As colitis (0.9%) and severe diarrhea (0.7%) may occur, patients should be carefully monitored.

4) Type 1 diabetes mellitus

As type 1 diabetes mellitus (including fulminant type 1 diabetes mellitus; 0.3%) may occur and lead to diabetic ketoacidosis, adequate attention should be paid to the development of symptoms, such as thirst, nausea, and vomiting. If type 1 diabetes mellitus is suspected, the administration should be discontinued, and appropriate measures such as insulin administration should be taken.

5) Immune thrombocytopenic purpura

As immune thrombocytopenic purpura (frequency unknown*) may occur, careful observation should be made, and if any abnormality is observed, the administration should be discontinued, and appropriate measures should be taken.

6) Hepatic dysfunction, hepatitis, sclerosing cholangitis

Hepatitis (0.2%), hepatitis (0.2%), and sclerosing cholangitis (frequency unknown*) with increased AST (GOT), ALT (GPT), γ-GTP, Al-P, and bilirubin may be observed, and if any abnormality is observed, the administration should be discontinued, or other appropriate measures should be taken.

7) Thyroid dysfunction

Thyroid dysfunction, such as hypothyroidism (5.6%), hyperthyroidism (1.2%), and thyroiditis (0.8%), may occur, so careful observation should be made, and appropriate measures such as discontinuation of administration should be taken if any abnormality is observed.

8) Neuropathy

Neuropathies, such as peripheral neuropathy (1.4%), polyneuropathy (0.1%), autoimmune neuropathy, Guillain-Barré syndrome, demyelination (frequency unknown*), and other neuropathy may occur; therefore, patients should be carefully monitored, and if any abnormality is observed, the administration should be discontinued, and other appropriate measures should be taken thing.

9) Renal failure

As renal insufficiency (0.4%), tubulointerstitial nephritis (0.2%), and other renal disorders may occur, renal function tests should be conducted periodically during the administration of this product, and appropriate measures such as discontinuation of administration should be taken of any abnormality is observed.

10) Adrenal disorders

As adrenal insufficiency (0.8%) and other adrenal disorders may occur, careful observation should be made, and if any abnormality is observed, the administration should be discontinued, or other appropriate measures should be taken.

11) Encephalitis

As encephalitis (frequency unknown*) may occur, careful observation should be made, and appropriate measures should be taken, including discontinuing drug administration if any abnormalities are observed.

12) Severe skin disorders

Severe skin disorders, such as toxic epidermal necrolysis (TEN), Stevens-Johnson syndrome (Stevens-Johnson syndrome) (incidence unknown*) and erythema multiforme (0.3%) may occur; therefore, patients should be carefully monitored. In this case, drug administration should be discontinued, and appropriate measures should be taken.

13) Venous thromboembolism

As venous thromboembolism, such as deep vein thrombosis (0.1%) and pulmonary embolism (0.1%), may occur, patients should be carefully monitored, and appropriate measures such as discontinuation of administration should be taken if any abnormalities are observed.

14) Infusion reaction

Infusion reactions (2.7%), including anaphylaxis, fever, chills, pruritus, rash, hypertension, hypotension, hypotension, dyspnea, or hypersensitivity, may occur. If a severe infusion reaction is observed, the administration should be discontinued immediately, appropriate measures should be taken, and the patient should be carefully monitored until these symptoms subside.

(3) Evaluation method and endpoints

1) Primary endpoint

Overall survival time

Secondary endpoint

Progression-free survival

Response rate

Health-related quality of life scores (EORTC QLQ-C30, EORTC QLQ-H&N35)

2) Observations/examinations

Patient Background

Basic Patient Information

Date of birth, age at the time of obtaining consent (automatically calculated from the date of birth and date of obtaining consent), sex, height at the time of obtaining consent, weight at the time of obtaining consent, body surface area at the time of obtaining consent (automatically calculated from height and weight), date of obtaining written consent

Target Disease Information

Histological diagnosis: squamous cell carcinoma

Primary sites: the maxillary sinus, oral cavity, nasopharynx, nasopharynx, oropharynx, hypopharynx, larynx

Pre-treatment Stage: III, IVA, IVB, IVC

TNM Classification: T (1, 2, 3, 4a, 4b), N (0, 1, 2, 3), M (0)

3) Inspection item

The following tests and observations should be performed periodically to evaluate drug safety and disease recurrence. However, in the event of an adverse event, additional laboratory tests or observations should be performed as needed, and the patient should be monitored until laboratory values or symptoms have resolved or abated. Details on the timing of each test and observation should be in accordance with "8.2 Schedule of Tests, Observations, and Reporting".

1) Hematological tests: hemoglobin, white blood cell count, neutrophil count, and platelet count

2) Blood biochemical tests: T.Bil, AST (GOT), ALT (GPT), Cr, Albumin, KL-6, SpO_2_, TSH, FT3, FT4, HbA1c, LDH*, ALP*, Na*, K*, Ca*, CRP*

Entries shall be made only for WBC, Neut, PLT, Hb, T.Bil, AST, ALT, Cr, KL-6, SPO_2_, TSH, FT3, FT4, HbA1c, and * shall be performed whenever possible

3) Ultrasound, CT, MRI, PET, histopathology, cytology, etc.

4) Tumor markers: SCC or CYFRA

5) PD-L1

6) Adverse events

Symptoms: fatigue (malaise), weight loss, skin rash/ desquamation, hyperpigmentation, loss of appetite, diarrhea, mucositis/stomatitis, nausea, vomiting, other (state as noted)

Grade: Indicates the highest grade for each case report period

Causation: yes, no

4) Lesion observation

Prior to the start of treatment and every 4 to 8 weeks of treatment, ultrasound, CT, MRI, and PET will be used to investigate the appearance of new lesions and other factors.

5) Health-related quality of life scores

The EORTC QLQ-C30 will be used as the baseline quality of life questionnaire used in malignant diseases, and the EORTC QLQ-H&N35 will be used as the disease-specific questionnaire. Quality of life questionnaires will be distributed to patients prior to the initiation of treatment and every 8 weeks after treatment initiation.

6) Subjective findings

Subjective symptoms and other findings should be monitored throughout the study period. Adverse events should be observed and examined as appropriate and, as a general rule, should be investigated until the event has resolved or the patient has returned to his or her pre-administration status, to the extent possible. This does not apply in the case of adverse events associated with the worsening of the primary disease.

The type and extent of adverse events should be in accordance with the NCI-CTC v4.0 JCOG version.

7) Follow-up items after termination (discontinuation)

Reason for termination (discontinuation), progress after discontinuation, and date of last observation

8) Time of data analysis

The survival of all patients will be investigated one year after enrollment of the last case after a total of 50 patients have been enrolled.

Once 20 patients have been enrolled, an interim evaluation and interim analysis will be conducted.

The interim analysis will also evaluate overall survival, progression-free survival, response rate, and health-related quality of life scores.

In the event of discontinuation due to adverse events, patients will be monitored until symptoms and laboratory values return to their pre-trial status, if possible, or until the investigating physician determines that further observation is no longer necessary, considering the safety of the patient.

9) Schedule of examinations, observations, and reports

|  | At the time of registration | Under treatment | | | End of treatment |
| --- | --- | --- | --- | --- | --- |
|  |  | Each course  starting time | Each course  Day 8 | At the time of discontinuation of treatment |  |
| Background/Agreement | ● |  |  |  |  |
| PS | ● | ● | ● | ● | ● |
| Medication status |  | ● | ● | ● | ● |
| Clinical testing | ● | ● | ● | ● | ● |
| QOL score | ● | Every 8 weeks |  |  |  |
| Subjective symptoms | ● | ● | ● | ● | ● |
| Tumor markers | ○ | ○ | ○ | ○ | ○ |
| Image evaluation | ● | Every 4 to 8 weeks | | | |

●：Required ○：To be performed as needed

10) Data collection

recording paper

The types of Case Report Forms (CRFs) to be used in the examination and their submission deadlines are as follows:

1) Registration Eligibility Form

The principal investigator will be contacted at the time of registration. The principal investigator will issue a registration number after confirming the information on the registration eligibility form and drug dosage.

2) Case Report Form

To be submitted after discontinuation or termination of study treatment.

3) Follow-up report.

　The survival and post-treatment of all patients will be investigated one year after the last case enrollment.

5. Study period

The study period will be conducted from after the ethical review approval until December 31, 2021.

The enrollment period will be conducted until December 31, 2019.

The observation period will be from after the ethical review approval until December 31, 2020.

The data analysis period will include the period from January 1, 2020, and will be conducted until December 31, 2021.

Patients enrolled in this study will continue to receive treatment within the scope of insurance if therapeutic effects are observed after the end of the enrollment period.

Translated with www.DeepL.com/Translator (free version)

6. Informed consent

(1) Explaining to patients

Prior to enrollment, the study physician will give the patient a narrative document, approved by the Medical Ethics Committee, and will verbally explain in detail the following

1. Overview of the study

2. Purpose of the test

3. Method of this test

4. The expected duration of patient participation in the study

5. The number of patients expected to participate in the study

6. Participation in the study is voluntary

7. If your health is compromised

8. Your privacy will be protected if the results of this test are made public

9. We will keep you posted on the test

10. The scientific and ethical validity of this study

11. Anticipated clinical benefits and risks or inconveniences

12. Sources of funding for this research

13. Payment of money and expenses to participants

14. Ownership of Intellectual Property Rights

15. Consent by the consignee

16. Your doctor

17. Please contact us at any time for advice

(2) Concurrence

After explaining the study to the patient and confirming that he or she fully understands, the patient is asked to participate in the study. The consent is of the patient's own free will. If the patient agrees to participate in the study, a consent form will be used, stating the name of the physician who explained, the name of the patient who received the explanation and consented, the date the consent was obtained, and a signature from both the physician and the patient. The consent explanation document and consent form (for the patient) should be given to the subject, and one copy should be kept in the medical record.

(3) Protection of privacy and patient identification

1) Adherence to the test protocol

Investigators participating in this study will comply with the protocol to the extent that patient safety and human rights are not compromised.

2) Changes to protocol content

The revised protocol must be approved by the principal investigator and the medical ethics committee before any changes can be made to the study protocol. However, this does not apply to medically unavoidable changes, such as those to avoid immediate danger to patients or changes that relate only to administrative matters of the study (e.g., a change in a telephone number).

7. Management of personal information

All parties involved in the study will strictly protect the personal information of the subjects in accordance with the Personal Information Protection Act. When the study physician provides case report forms, adverse events, and other relevant data outside of this institution, adequate care will be taken to protect personal information to prevent third parties from identifying the subject, such as the use of subject identification codes to describe the subject. Identification and reference of registered subjects will be made using the registration number issued at the time of registration. The subjects’ personal information will be protected in the same way when the results of this study are made public.

8. The burden, anticipated risks, and benefits to research subjects, a comprehensive assessment of these risks and benefits, and measures to minimize such burden and risks.

(1) Projected profit

Nivolumab has the potential to extend the time to recurrence and survival. Nivolumab may also improve health-related quality of life scores.

(2) Anticipated burden and risks

Nivolumab is known to produce an excessive immune response and may cause an excessive immune response to T-cell activation.

(3) Overall evaluation of benefits and disadvantages and countermeasures for disadvantages

Cetuximab-based chemotherapy (cisplatin, 5-FU, and cetuximab) and nivolumab are the branches of choice with the highest level of evidence for the treatment of head and neck cancer with recurrence or distant metastases. Cetuximab-based chemotherapy requires 6 courses of 3 weeks per course and requires prolonged hospitalization, and the combination of the three drugs has a high incidence of adverse events. Nivolumab is administered every 2 weeks, which means shorter hospital stays and no chemotherapy-specific adverse events, but there is the potential for adverse events specific to the sparing agent.

Although platinum-based chemotherapy is recommended for head and neck cancers with recurrence or distant metastases, there are no treatment options that have prolonged survival in patients. Nivolumab is the first drug to demonstrate an overall survival (OS) benefit in head and neck cancers with recurrent or distant metastases that have been previously treated with chemotherapy; therefore, the benefit is considered high in terms of risk-benefit considerations.

9. Storage and disposal of samples and information (including materials pertaining to information used in research)

Important documents related to the conduct of the research and other documents or records necessary to assure the reliability of the data shall be retained until the date five years have elapsed since the cessation or completion of the research and then disposed of with care for personal information.

10. Contents and methods of reporting to the head of the research institution

Report the progress of the research to the President at least once a year. Reports on the discontinuation or termination of research shall be made promptly on each occasion.

11. Conflicts of interest regarding the source of funding, the research institution, and the researchers, including individual earnings

This is a single-center Phase II study, and the treatment to be conducted in this study will be administered within the normal course of medical care. In the planning, conduct, and reporting of the study, we will ensure that there are no "possible conflicts of interest" that may affect the results of the study and the interpretation of the results and that the conduct of the study will not interfere with the rights and interests of the subjects.

The study will be funded by the principal investigator's home department. Conflict of interest (COI) in the principal investigator and the research office will be as follows:

Neither the principal investigator nor the secretariat has any conflicts of interest related to the study that should be disclosed.

12. Method of disclosure of research-related information

The principal investigator will register an outline of the study in the University Hospitals Medical Information Network (UMIN-CTR) prior to conducting the study and will update the research protocol as needed, as well as making any changes to the research protocol and the progress of the study. When the research is completed, the results of the study shall be registered without delay. When the results are made public, the researcher shall take the necessary measures to protect the rights of the research subjects and their related persons or the rights and interests of the researcher and their related persons. The researcher shall make public the results of this research by presenting them at relevant conferences.

13. Responses to consultations with research subjects and related parties

Principal Investigator: Kiyoaki Tsukahara, MD

Department of Otorhinolaryngology, Head and Neck Surgery, Tokyo Medical University

6-7-1 Nishi-Shinjuku, Shinjuku-ku, Tokyo 160-0023, Japan

Tel: +81-3-3342-6111 (representative)

Research Office (contact person)

Department of Otorhinolaryngology, Head and Neck Surgery, Tokyo Medical University

6-7-1 Nishi-Shinjuku, Shinjuku-ku, Tokyo 160-0023, Japan

Tel: +81-3-3342-6111 (representative)

Sub-investigator:

Isaku Okamoto, MD

Hiroki Sato, MD

14. If there is an economic burden or honorarium on the research subjects, if any, and its details

not applicable

15. Invasion (excluding minor invasion). In the case of research that involves invasion (except for minor invasion), what to do in the event of a serious adverse event:

(1) Adverse events and safety assessment

The primary analysis population for adverse events is the analysis of cases in which protocol treatment is initiated.

(2) Adverse events and side effects

An adverse event is any unfavorable or unintended sign (including abnormal changes in laboratory values), symptom, or disease that occurs in a subject to whom the study drug is administered, whether or not causally related to the study drug.

Adverse events that are "reasonably causally related to the study drug" (other than "not reasonably causally related to the study drug") should be treated as adverse reactions.

The Common Terminology Criteria for Adverse Events v4.0 (CTCAE v4.0) should be used to evaluate adverse events and make a grade.

In this study, a grade worsening of one or more grades on CTCAE v4.0 is considered an adverse event. Abnormalities in laboratory test results are also considered to be adverse events. A grade worsening of one or more levels of CTCAE v4.0 should be reported as an adverse event, even if the event was occurring prior to treatment.

(3) Serious adverse event means any of the adverse events specified in "Adverse Events and Adverse Reactions" and includes the following:

1) Anything that leads to death

2) Any permanent or significant disability or dysfunction

3) A threat to life

4) When hospitalization or prolonged hospitalization is required for treatment

5) Other medically significant conditions

6) Congenital anomalies

(4) Causal relationships with test agents in adverse events

The determination of causation in adverse events is based on consideration of the subject's general condition, comorbidities, concomitant medications and concomitant therapies, and temporal relationships. There are two types of causal relationships: "There is a reasonable causal relationship with the study drug" and "There is no reasonable causal relationship with the study drug.

(5) Response to adverse events

If an adverse event is observed, the investigating physician will take appropriate action against the subject and report it in the case report form. Patients who continue to experience adverse events should be followed up until they have recovered to the greatest extent possible. Adverse events (causally related to the study drug) should be monitored to the extent possible. However, this does not apply in cases where symptoms are chronic due to worsening of the primary disease or worsening of complications, or when it is difficult to observe the patient due to transfer to a hospital or initiation of post-treatment therapy.

(6) Response to serious adverse events

In the event of a serious adverse event, the principal investigator should be informed of the seriousness of the event in accordance with the Rapid Response Procedure and response.

(7) Rapid response procedure and actions for rapid response reports

In the event of an adverse event that is the subject of a rapid transport report, regardless of whether the event is causally related to the treatment, the study investigator will report the event to the principal investigator in person or by telephone within 24 hours. The principal investigator will determine the urgency, importance, and impact of the report, and if necessary, will take measures such as suspending enrollment and urgently notifying the participating institutions of the event.

(8) Criteria for study termination

In the following cases, enrollment will be terminated, and the research organization and, if necessary, the ethics committee will review whether or not the research can be continued, and if it is deemed appropriate to terminate the research itself, the research will be terminated immediately:

1) In the event of treatment-related death

2) In the event of an unexpected serious adverse event

16. Whether there is compensation for health hazards resulting from the research and the details of that compensation:

If any adverse events occur as a result of this study and the subject's health is affected, the principal investigator, sub-testing physician, and the medical institution will provide the best possible care, including appropriate treatment and other necessary measures.

Because the drug used in this study is a drug approved by insurance for the treatment of head and neck cancers, in the event of health problems, the best possible treatment, including necessary medical treatment, will be provided. Still, this treatment will be provided by insurance, and no monetary compensation will be provided.

17. In the case of research that involves medical treatment that goes beyond the scope of ordinary medical care, measures regarding the provision of medical care to research subjects after the research is conducted:

Not applicable

18. If the research is likely to yield important findings regarding the health of the research subject, genetic characteristics that may be passed on to the progeny, etc., the research results on the research subject (including incidental findings) will be handled. Handling of the research results on research subjects (including incidental findings):

Not applicable

19. In the event that a part of the work related to the research is consigned, the nature of the work and the method of supervision of the consignee:

Not applicable

20. If there is a possibility that samples and information obtained from research subjects may be used for future research that is not specified at the time of receiving consent from the research subjects, etc., or that may be provided to other research institutions, this fact and what is anticipated at the time of receiving consent:

Not applicable

21. In the event that monitoring and auditing are to be carried out, the implementation system and procedures are as follows:

In this study, regular monitoring will be conducted twice a year to ensure that the research is being conducted safely and according to protocol and that data are being collected accurately. The research secretariat will conduct monitoring based on the data entered in the CRF and will take the lead in this monitoring. The purpose of periodic monitoring is to provide feedback on problem areas and to improve the scientific ethics of the research. Audits will be conducted as necessary.

Items to be monitored:

1) Registration status: number of registrations - cumulative/period, by

2) Eligibility: ineligible cases/potentially ineligible patients

3) Pre-treatment background factors

4) Whether a patient is on or off protocol treatment, and the reason for discontinuation or termination

5) Protocol deviation

6) Serious adverse events

7) Adverse reactions/adverse events

8) Other issues related to the progress of the study and safety

22. Implementation system

Principal investigator: Kiyoaki Tsukahara, MD

Department of Otorhinolaryngology, Head and Neck Surgery, Tokyo Medical University

6-7-1 Nishi-Shinjuku, Shinjuku-ku, Tokyo 160-0023, Japan

Tel: +81-3-3342-6111 (representative)

Research Office (contact person)

Department of Otorhinolaryngology, Head and Neck Surgery, Tokyo Medical University

6-7-1 Nishi-Shinjuku, Shinjuku-ku, Tokyo 160-0023, Japan

Tel: +81-3-3342-6111 (representative)

Sub-investigator:

Isaku Okamoto, MD

Hiroki Sato, MD

**Supplementary Table 1. Changes in quality-of-life scores**

|  | **QOL score**  **LS mean (95% CI)** | **Change from baseline**  **LS mean (95% CI)** | ***P*-value**  **for all** | ***P*-value**  **vs. baseline** |
| --- | --- | --- | --- | --- |
| **Functional scales** |  |  |  |  |
| **Physical functioning** |  |  | 0.349 |  |
| **Pre-nivolumab** | 79.6 (66.6–92.7) |  |  |  |
| **8 weeks** | 78.9 (65.9–92.0) | -0.7 (-5.9–4.5) |  | 0.787 |
| **16 weeks** | 75.5 (62.3–88.8) | -4.1 (-10.0–1.8) |  | 0.165 |
| **Role functioning** |  |  | 0.381 |  |
| **Pre-nivolumab** | 72.8 (54.5–91.1) |  |  |  |
| **8 weeks** | 73.7 (55.4–92.0) | 0.9 (-16.9–18.7) |  | 0.921 |
| **16 weeks** | 61.0 (40.9–81.1) | -11.8 (-31.6–8.0) |  | 0.234 |
| **Emotional functioning** |  |  | 0.686 |  |
| **Pre-nivolumab** | 82.9 (73.5– 92.3) |  |  |  |
| **8 weeks** | 85.1 (75.7–94.5) | 2.2 (-4.3–8.7) |  | 0.497 |
| **16 weeks** | 82.3 (72.4–92.2) | -0.6 (-7.9–6.7) |  | 0.862 |
| **Cognitive functioning** |  |  | 0.825 |  |
| **Pre-nivolumab** | 78.9 (65.0–92.9) |  |  |  |
| **8 weeks** | 77.2 (63.2–91.1) | -1.8 (-9.0–5.5) |  | 0.624 |
| **16 weeks** | 76.7 (62.4–91.1) | -2.2 (-10.3–5.9) |  | 0.581 |
| **Social functioning** |  |  | 0.493 |  |
| **Pre-nivolumab** | 82.5 (73.1–91.8) |  |  |  |
| **8 weeks** | 88.6 (79.3–97.9) | 6.1 (-4.3–16.6) |  | 0.239 |
| **16 weeks** | 85.8 (75.3–96.3) | 3.3 (-8.2–14.9) |  | 0.559 |
| **Global health status** |  |  |  |  |
| **Global health status/QoL** |  |  | 0.762 |  |
| **Pre-nivolumab** | 65.4 (53.8–76.9) |  |  |  |
| **8 weeks** | 64.0 (52.5–75.6) | -1.3 (-9.2–6.5) |  | 0.735 |
| **16 weeks** | 67.2 (55.1–79.3) | 1.9 (-6.9–10.7) |  | 0.666 |
| **Domain scales** |  |  |  |  |
| **Pain** |  |  | 0.332 |  |
| **Pre-nivolumab** | 8.3 (3.1–13.6) |  |  |  |
| **8 weeks** | 11.4 (6.1–16.7) | 3.1 (-2.0–8.1) |  | 0.223 |
| **16 weeks** | 12.0 (6.2–17.8) | 3.6 (-2.0–9.2) |  | 0.195 |
| **Swallowing** |  |  | 0.424 |  |
| **Pre-nivolumab** | 18.9 (7.4–30.3) |  |  |  |
| **8 weeks** | 23.2 (11.8–34.7) | 4.4 (-3.6–12.4) |  | 0.274 |
| **16 weeks** | 23.9 (11.9–35.9) | 5.0 (-3.9–14.0) |  | 0.261 |
| **Sense problems** |  |  | 0.342 |  |
| **Pre-nivolumab** | 19.3 (5.5–33.1) |  |  |  |
| **8 weeks** | 19.3 (5.5–33.1) | 0.0 (-10.5–10.5) |  | > 0.999 |
| **16 weeks** | 27.0 (12.3–41.6) | 7.7 (-4.1–19.4) |  | 0.192 |
| **Speech problems** |  |  | 0.237 |  |
| **Pre-nivolumab** | 22.2 (10.7–33.7) |  |  |  |
| **8 weeks** | 26.3 (14.8–37.8) | 4.1 (-5.6–13.8) |  | 0.395 |
| **16 weeks** | 31.4 (19.0–43.8) | 9.2 (-1.6–20.0) |  | 0.093 |
| **Trouble with social eating** |  |  | 0.956 |  |
| **Pre-nivolumab** | 28.9 (18.5–39.4) |  |  |  |
| **8 weeks** | 28.5 (18.1–38.9) | -0.4 (-8.3–7.4) |  | 0.910 |
| **16 weeks** | 27.7 (16.6–38.7) | -1.3 (-10.1–7.5) |  | 0.767 |
| **Trouble with social contact** |  |  | 0.484 |  |
| **Pre-nivolumab** | 14.7 (6.2–23.3) |  |  |  |
| **8 weeks** | 14.0 (5.5–22.6) | -0.7 (-8.2–6.8) |  | 0.849 |
| **16 weeks** | 10.0 (0.7–19.3) | -4.7 (-13.1–3.6) |  | 0.254 |
| **Less sexuality** |  |  | 0.728 |  |
| **Pre-nivolumab** | 28.9 (12.4–45.5) |  |  |  |
| **8 weeks** | 24.6 (8.0–41.2) | -4.4 (-16.1–7.4) |  | 0.452 |
| **16 weeks** | 28.2 (10.7–45.7) | -0.8 (-13.9–12.4) |  | 0.906 |

QOL, quality of life; LS mean, least square mean; 95% CI, 95% confidence interval
